# Novel Miniature and Selective Combustion-Type CMOS Gas Sensor for Gas-Mixture Analysis—Part 1: Emphasis on Chemical Aspects

**DOI:** 10.3390/mi11040345

**Published:** 2020-03-26

**Authors:** Dima Shlenkevitch, Sara Stolyarova, Tanya Blank, Igor Brouk, Yael Nemirovsky

**Affiliations:** Electrical Engineering Department, Technion—Israel Institute of Technology, Haifa 32000, Israel; ssstolya@technion.ac.il (S.S.); tblank@technion.ac.il (T.B.); bigor@tx.technion.ac.il (I.B.); nemirov@ee.technion.ac.il (Y.N.)

**Keywords:** catalytic gas sensor, CMOS–SOI–MEMS, selectivity, Pt nanoparticle, inkjet printing

## Abstract

There is an ongoing effort to fabricate miniature, low cost, sensitive, and selective gas sensors for domestic and industrial uses. This paper presents a miniature combustion-type gas sensor (GMOS) based on a thermal sensor, where a micromachined CMOS–SOI transistor integrated with a catalytic reaction plate acts as a sensing element. This study emphasizes GMOS performance modeling, technological aspects, and sensing-selectivity issues. Two deposition techniques of a Pt catalytic layer suitable for wafer-level processing were compared, magnetron sputtering and nanoparticle inkjet printing. Both techniques have been useful for the fabrication of GMOS sensor, with good sensitivity to ethanol and acetone in the air. However, a printed Pt nanoparticle catalyst provides almost twice as much sensitivity as compared to that of the sputtered catalyst. Moreover, sensing selectivity in the ethanol/acetone gas mixture was demonstrated for the GMOS with a Pt nanoparticle catalyst. These advantages of GMOS allow for the fabrication of a low-cost gas sensor that requires a low power, and make it a promising technology for future smartphones, wearables, and Internet of Things (IoT) applications.

## 1. Introduction

In recent years, the technological trend in the field of gas sensors has tended toward miniaturized and low-cost CMOS-compatible technologies [1,2,3,4]. Currently, metal oxide semiconductor (MOX) gas sensors are dominating the field of commercial miniature gas sensors. These sensors measure resistance changes of sensing material because of the change of the electronic structure caused by the reaction of absorbed oxygen with the gas [5,6,7,8]. This technology has several advantages, such as good sensitivity and simplicity in measuring a response signal. However, it suffers from long-term instability, environmental influence, and poor selectivity.

Another technology for miniature gas sensors uses temperature-change measurements as a working principle. These sensors, known as pellistors, measure thermal changes caused by an exothermic gas-combustion reaction occurring on a heated catalytic layer, which activates the reaction [9,10,11]. The thermal sensor is a key element in these gas sensors. It is usually a resistor, but thermopiles and pyroelectric ceramics were also reported [12,13,14].

**Figure 1:**
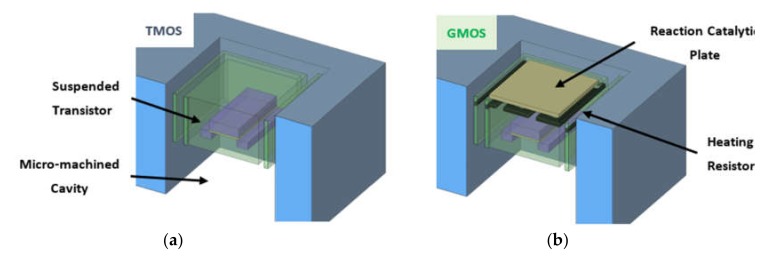
Micromachined cavity of (**a**) thermal sensor (TMOS) and (**b**) gas sensor (GMOS) based on TMOS sensor.

In the last decade, a thermal sensor (TMOS) based on CMOS–SOI technology was studied [15,16,17,18,19,20,21]. TMOS is a type of thermal sensor that detects temperature changes induced by IR radiation absorption. The sensing element is a suspended MOSFET transistor (Figure 1a) that operates at subthreshold, and therefore requires low power consumption. IR radiation increases transistor temperature and modifies current–voltage (I–V) characteristics. A TMOS transistor released by MEMS/NEMS processing represents an uncooled thermal sensor with high temperature sensitivity in terms of the temperature coefficient of voltage (TCV[1/K]=(dV/dT)/V), it requires low power, and is fabricated at low cost.

These TMOS advantages were applied to develop a combustion-type gas sensor (GMOS). To fabricate the GMOS, an integrated heating resistor was added to a TMOS thermal sensor (Figure 1b), and the catalytic layer was applied on the top- or back-side surface of the pixel. The feasibility of GMOS sensing of ethanol and acetone was proven in [22,23]. In contrast to MOX sensors where the sensing element directly interacts with the gas, the GMOS sensing element (the transistor) does not; therefore, the sensor has potential for long-term stability.

The exothermic gas-combustion reaction occurs on the surface of the catalytic layer. Its chemical and structural stability, and its adhesion to the transistor stage, are critical for the reliable operation of a GMOS sensor. The well-known phenomenon of catalyst deactivation related to mechanical, thermal, or chemical aspects may influence combustion-reaction efficiency and sensor performance [24]. However, it can be postponed or avoided by proper material selection and refreshment technology.

Therefore, in the experiment part of the paper, we focused on the development of deposition technology and the post-treatment of the catalytic layer. The catalyst was deposited onto the pixel using two techniques, magnetron sputtering and nanoparticle inkjet printing. Both were applied as a back-end process for released sensors after MEMS processing, which makes it challenging to leave thin MEMS membranes undamaged. Another key factor in deposition-technique selection was the ability to manufacture these sensors at wafer-level processing in order to reduce the total fabrication cost, which is critical for high-volume commercial applications such as mobiles, wearables, and Internet of Things (IoT) sensor markets.

GMOS performance modeling is presented here on the basis of chemical and thermal considerations. The proof of concept for selective sensing in gas mixtures is demonstrated for a GMOS with a Pt nanoparticle catalytic layer.

## 2. Materials and Methods

### 2.1. GMOS Design and Operation Principle

GMOS is a tiny combustion-type gas sensor based on the thermal TMOS sensor. The sensing transistor detects temperature changes induced by heat that is released by the exothermic chemical reaction of gas combustion on a catalytic layer. The temperature change modifies I–V characteristics of the sensing transistor operated at subthreshold. Sensing is based on differentially measuring either voltage or current changes at the applied operation point of the transistor. In order to activate the chemical reaction, the pixel with the catalytic layer is heated by an embedded heating resistor made of tungsten.

**Figure 2:**
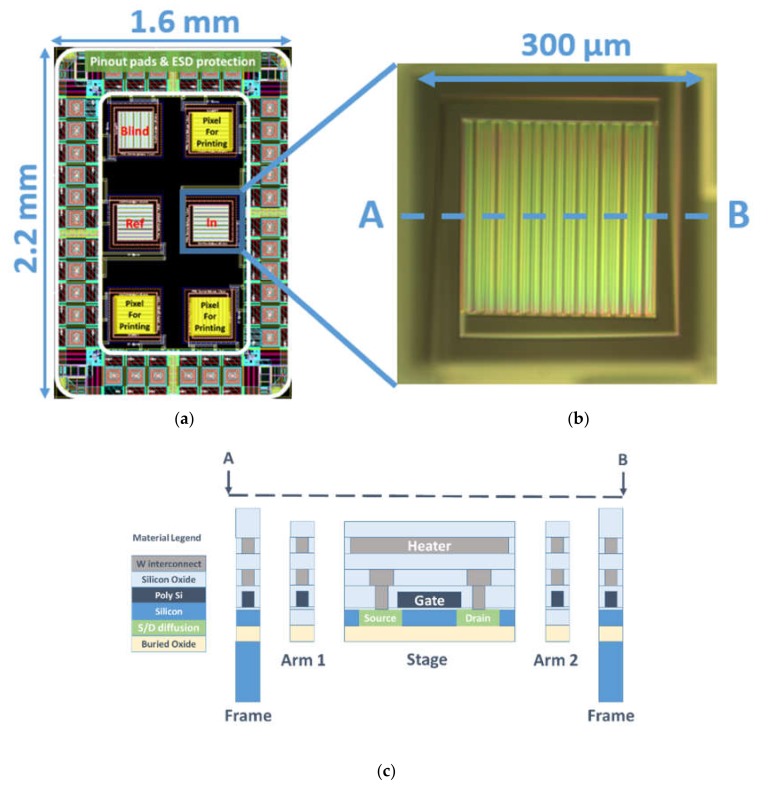
Overview of typical GMOS sensor die and pixel construction: (**a**) GMOS die layout containing three pixels for catalytic layer, one reference (“Blind”) and two auxiliary pixels (“In” and “Ref”) for stabilizing operational point of the electrical readout [22]; (**b**) optical image of released transistor pixel without catalytic layer; (**c**) cross section on plane A–B of released pixel.

The overview of the GMOS die, the single pixel, and its cross section is shown in Figure 1 and Figure 2. The GMOS die, with an overall area of 1.6 × 2.2 mm, contained six identical 300 × 300 µm micromachined pixels. Three of them were the active pixels, covered with the same or different catalytic layers; one pixel was a “blind” pixel for differential temperature measurement, and the two others were needed for stabilizing the operational point of the electrical readout. The voltage signal caused by a temperature change on each active pixel was separately measured relative to the blind pixel. Differential measurements needed to exclude transistor self-heating effects and ambient-temperature variations. The electronic-circuitry and differential-readout concepts are reported in [22].

Each pixel contained a built-in heating resistor and an effective transistor, as shown in Figure 2c. The effective transistor, covering the entire stage area of 213 × 213 µm, was a combination of 29 transistors with L = 4.15 µm and W = 204 µm, connected in parallel. A heating resistor of 300 Ω nominal value heated the stage in pulsed mode with a duty cycle of 50%. Total heating-power consumption of the die was about 10–15 mW, which is comparable to commercial gas sensors [25,26]. Transistor power consumption was relatively negligible because of the subthreshold operation.

### 2.2. Experiment Gas Chamber

**Figure 3:**
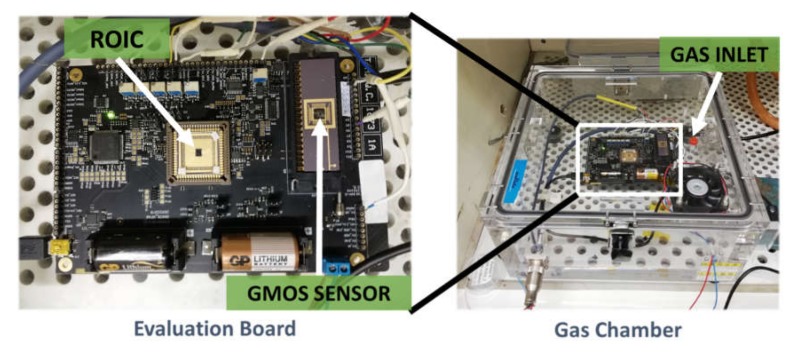
Experiment setup.

The experiment setup, shown in Figure 3, included a sealed gas chamber of 6 L volume and an evaluation board (EVB). The battery-powered evaluation board was computer-controlled via USB, and included the GMOS sensor and readout circuit, both bonded to the chip carriers. The analyte gas could be introduced into the chamber in gaseous or liquid form as a drop through the gas inlet. When a liquid drop was introduced to the chamber, the measurement signal was accepted after full drop evaporation. The gas concentration inside the chamber was calculated using the gas-law equation.

### 2.3. Catalyst-Deposition Technology

**Figure 4:**
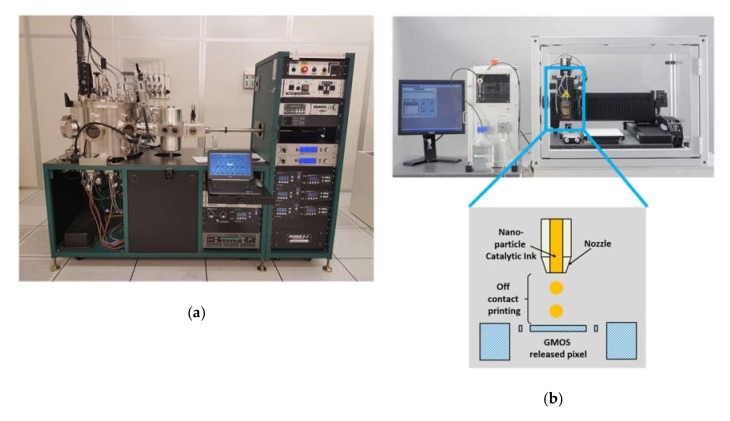
Two deposition systems used in this study: (**a**) magnetron sputtering system ATC 2200 [27]; (**b**) inkjet printer sciFLEXARRAYER S3 [28] with off-contact-printing schematic.

This work focused on two techniques for catalytic-layer deposition, magnetron sputtering [27] and inkjet printing [28]; both deposition systems are shown in Figure 4. The technological criteria for the optimal technique choice are as follows: (i) Compatibility with wafer-level processing, (ii) contactless deposition, (iii) precise deposition on specific pixels, and (iv) postprocessing without additional masking. However, these are the catalytic properties of the resulting film that play the decisive role in the final choice of the technology.

The catalytic layer was applied as a back-end process on the released micromachined pixels. Magnetron sputtering was chosen as a well-established technique compatible with CMOS wafer-level processing. Additional masking with a mechanical stencil mask was required for sputtering to prevent deposition on the blind, operational pixels, and pads (in the case of front-side deposition). A thin Pt layer of 2000 Å was in situ sputtered on top of the Ti thin sputtered layer of 30 Å. The Ti film was used as an adhesion layer to prevent catalytic-layer cracking or delamination during heating cycles, and to thus improve its mechanical stability.

Inkjet printing is a relatively novel technique for micromachined devices, allowing the use of nanoparticle ink. Nanoparticle catalysts are expected to provide a higher effective area and higher activity [29] in comparison to thin-film catalysts deposited by sputtering; therefore, a higher sensor signal is expected. Among other advantages of this printing technique are compatibility with wafer level processing, no need for masking, low consumption of precious materials, and lower system price in comparison with thin-film deposition methods. It is indeed a challenging process to deposit a catalytic layer on a released transistor stage after MEMS processing without additional masking. Printing should be done without any contact between printer head and transistor stage surface. Another printer demand was positioning accuracy, as the stage area was about 213 × 213 µm, and reasonable printer accuracy is required for precise layer deposition.

**Figure 5:**
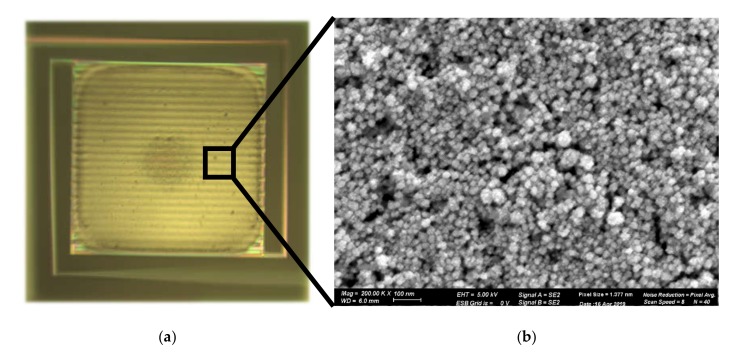
Pt nanoparticle catalytic-layer characterization: (**a**) Optical image of pixel with catalyst printed on top of it; (**b**) SEM micrograph of catalytic layer.

Inkjet-printer model Scienion sciFLEXARRAYER S3 that was used in this work met the above requirements. For this printer, commercial water-based ink with Pt nanoparticles (20 wt %) was purchased from Fraunhofer Institute, Dresden [30]. After ink-printing, the GMOS die was annealed at 280 °C on a hot plate for 1 h to evaporate the ink solvents. The obtained printed Pt nanoparticle layer of about 0.5 µm thickness is shown in Figure 5. As an effective reaction area is important for better sensitivity, maximal coverage of the pixel area is key. Figure 5a shows that pixel coverage with the catalytic layer was close to the net pixel area, and that printing precision was adequate. The layer exhibited a nanostructure with a typical nanoparticle size of 10–25 nm (Figure 5b).

The sensor dies presented in this paper enable heating pixels to relatively high temperatures (above 350 °C) by using the embedded heater, and make it possible to perform catalyst refreshment. Because of high-temperature heating in an air environment, the residual products of combustion reactions and other contaminants, such as siloxane, can be removed from the catalytic surface. The sensing layer was preheated with the integrated heater for several minutes to temperatures above 350 °C prior to measurements. In addition, periodic short heating was applied for several seconds between measurements. These procedures indeed showed significant improvement in signal stability and an ability to recover signal degradation.

## 3. Results and Discussion

### 3.1. GMOS Performance Model

GMOS is a thermal-gas sensor that operates at elevated temperatures to activate the combustion of analyte gas on the reaction catalytic layer. Each GMOS pixel has a built-in resistor that increases catalytic-layer temperature by joule heating dissipation. The heat-flow equation could be written as follows.

(1) $$C_{TH}\frac{d\left(T-T_{0}\right)}{dt}+G_{TH}\left(T-T_{0}\right)=P_{Joule-Heating}+P_{Reaction},$$

where *T*_0_ is ambient temperature; *C_TH_* (J/K) and *G_TH_* (W/K) are sensor thermal capacitance and conductance, respectively; *P_Joule-Heating_* (W) and *P_Reaction_* (W) are joule heating power and exothermic oxidation reaction power, respectively.

In a steady state, Equation (1) is reduced to

(2) $$T=T_{0}+\frac{P_{Joule-heating}}{G_{th}}+\frac{P_{Reaction}}{G_{th}}=T_{0}+\Delta T_{Joule-Heating}+\Delta T_{Reaction}.$$

Therefore, the temperature of the catalytic layer is a sum of ambient temperature, temperature increase due to joule heating, and temperature increase due to the gas-combustion exothermic reaction.

To obtain the heating-resistor value, it was required to determine the temperature increase due to joule heating in the absence of a chemical reaction:

(3) $$\Delta T_{Joule-Heating}=\frac{P_{Joule-Heating}}{G_{TH}}=\frac{I\cdot V}{G_{TH}}=\frac{I^{2}\cdot R(T)}{G_{TH}}=\frac{V^{2}}{R(T)\cdot G_{TH}},$$

where *R(T)* is the value of GMOS built-in resistor that heats the reaction catalytic layer. By knowing the needed working temperature, Equation (3) allows to set the needed resistor value for the required temperature.

Output signal v_sig_ (V) is the difference between drain-source voltages of transistors *V_DS_* in active and blind pixels [22]. Ambient temperature and temperature increase because joule heating are equal for blind and active pixels; therefore, output signal is directly related to the temperature increase obtained from the power released by the exothermic chemical reaction of the analyte gas:

(4) $$v_{signal}=\left(\frac{dV_{DS}}{dT}\right)\cdot \Delta T_{Reaction}=\left(\frac{dV_{DS}}{dT}\right)\cdot \left(\frac{P_{Reaction}}{G_{TH}}\right)$$

The power of the exothermic oxidation reaction was modeled as the flux of consumed molecules of analyte gas on catalytic surface (*F_Surface_*; molecules/m^2^s) multiplied by the effective area of the catalytic layer (area; m^2^) and combustion enthalpy (∆*H_C_*; joule/mole) divided by the Avogadro number (*N_A_*):

(5) $$P_{Reaction}=F_{Surface}\cdot Area\cdot \left(\frac{\Delta H_{C}}{N_{A}}\right).$$

The flux of consumed molecules at surface reaction (for first-order reaction) can be expressed as

(6) $$F_{Surface}=k_{S}C_{S}=Z\exp(-\frac{E_{A}}{k_{B}T})\cdot C_{S},$$

where *k_S_* is the reaction rate (m/s); *Z* is the Arrhenius constant (m/s); *E_A_* is the activation energy for combustion reaction (joule/mole); and *C_S_* is the gas concentration at catalytic surface (molecules/m^3^).

The flux of molecules that arrive to the catalytic surface was modeled as diffusion from bulk gas to the catalytic surface through a stagnant film:

(7) $$F_{Diffusion}=D\cdot \frac{\left(C_{G}-C_{S}\right)}{\delta },$$

where *D* is the gas diffusion constant (m^2^/s); *δ* is the stagnant film thickness (m); and $C_{G}=n_{Gas}/V=[P/kT]\cdot C_{ppm}\cdot 10^{-6}$ is the ambient gas concentration (molecules/m^3^). *P* and *T* are the ambient pressure and temperature, respectively. *C_ppm_* is the gas concentration in ppm = 10^−6^ air molecules.

In a steady-state condition, there is equilibrium between the fluxes of consumed and arrived molecules by diffusion, *F_Diffusion_* = *F_Surface_* = *F*. Therefore, gas concentration at the reacting surface can be expressed in terms of ambient gas concentration. The gas flux toward the reacting surface is governed by Equation (8).

(8) $$F=F_{Surface}=F_{Diffusion}=C_{G}\cdot {\left(\frac{1}{k_{S}}+\frac{\delta }{D}\right)}^{-1}.$$

**Figure 6:**
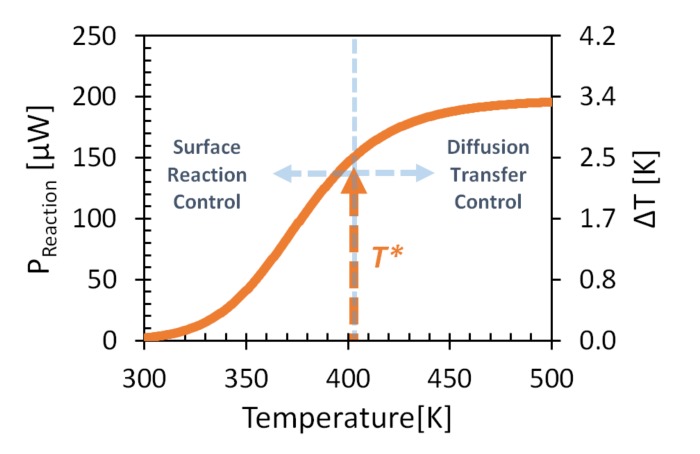
Calculated chemical-oxidation reaction power release due to 100 ppm combustion of ethanol on Pt catalyst as function of heated-pixel temperature. Average *G_TH_* = 60 µW/K, *D*/*δ* = 1 m/s [22], *E_A_* ≈ 55.23 kJ/mole, and *Z* ≈ 10^7.66^ m/s [32] were assumed.

Modeling the power released by the reaction gives sigmoid-like temperature dependence (Figure 6) [31]. At low temperatures, small heat generation was observed that exponentially increased with increasing temperature until saturation at high temperatures. The low-temperature range corresponded to surface reaction control, and the high-temperature range corresponded to gas-diffusion transfer control. The ignition temperature (denoted below by T*) is defined as the transition temperature between the two regimes. The ignition temperature relates to the maximal change of released power as a function of temperature, and it is specific for the analyte-gas and catalytic-layer combination.

This final-output voltage-signal model takes into consideration GMOS’ electrical, thermal, and chemical properties:

(9) $$v_{signal}=\left(\frac{dV_{DS}}{dT}\right)\cdot C_{G}\cdot {\left(\frac{1}{k_{S}}+\frac{\delta }{D}\right)}^{-1}\cdot Area\cdot \left(\frac{\Delta H_{C}}{G_{TH}\cdot N_{A}}\right).$$

The voltage signal is linearly dependent on gas concentration, the effective area of the catalytic layer, and sensitivity at a defined operational point of the transistor. Therefore, the Pt nanoparticle catalyst was expected to increase the effective area and improve the output signal.

Ignition temperature T* is a characteristic temperature for the gas and catalytic layer, and could therefore be used as the selectivity term. For selective gas sensing, chemical sensitivity (*S_Chemical_*; V/ppm) needs to be defined, as is shown in Equation (10).

(10) $$S_{Chemical}=\frac{v_{signal}}{C_{G}}=\left(\frac{dV_{DS}}{dT}\right)\cdot {\left(\frac{1}{k_{S}}+\frac{\delta }{D}\right)}^{-1}\cdot Area\cdot \left(\frac{\Delta H_{C}}{G_{TH}\cdot N_{A}}\right)$$

The chemical-sensitivity term is defined for analyte gas at a characteristic ignition temperature for this gas and the catalytic layer.

### 3.2. Sensitivity

**Figure 7:**
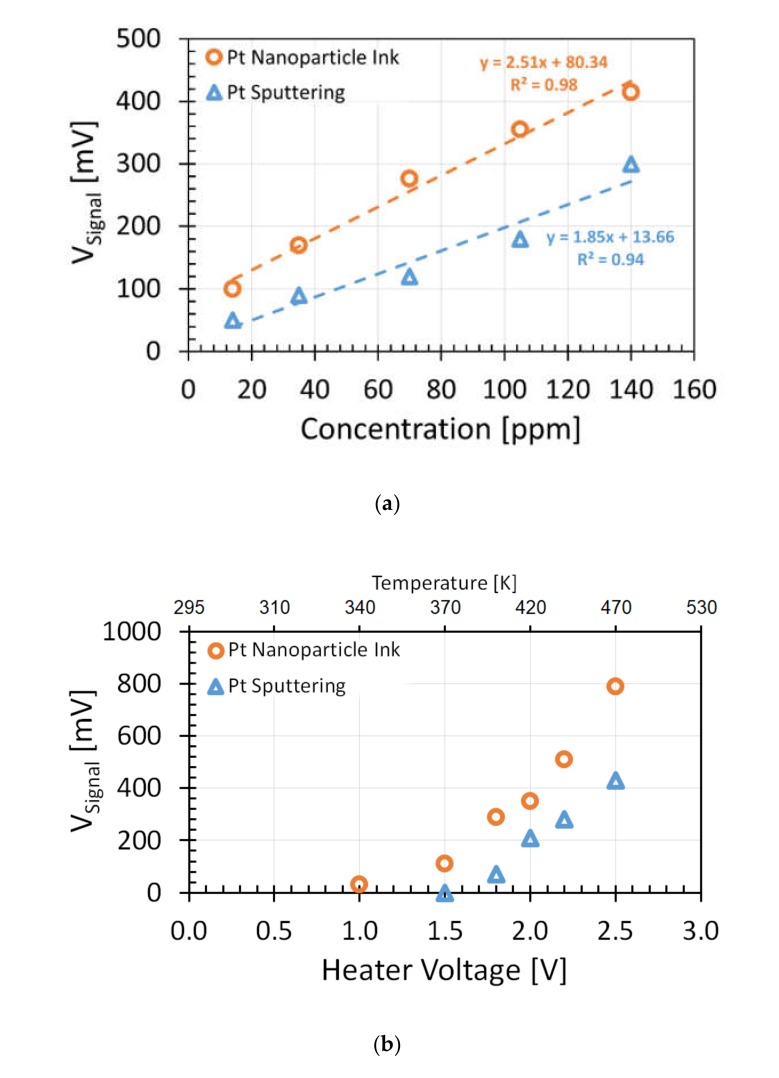
Experiment results for ethanol detection over Pt nanoparticle layer and sputtered Pt thin film layer: (**a**) Voltage signal as function of ethanol concentration at constant heater voltage of 2 V; (**b**) voltage output signal as a function of heater voltage applied to heating resistor at constant temperature and at constant concentration of 100 ppm of ethanol. Voltage output signal with 5.2 dB gain.

To compare the sensitivity of GMOS sensors with a sputtered and printed Pt catalyst to ethanol, the concentration dependencies of the sensor signals at a constant pixel temperature were measured. Experiment results showed a linear response of GMOS to ethanol gas (Figure 7a) for both catalytic layers in the range of 10–140 ppm with a high sensitivity of 2.5 and 1.8 mV/ppm at a gain of 5.2 dB for the Pt nanoparticle layer and sputtered Pt thin film, respectively. As expected, the GMOS with printed nanoparticle catalyst showed higher efficiency.

Accordingly, voltage signal as a function of heating voltage applied to the heating resistor at constant gas concentration was also higher for the Pt nanoparticle catalytic layer in comparison with the Pt sputtered layer (Figure 7b). This can be explained by nanostructured surface morphology of the Pt nanoparticle catalyst, as shown in Figure 5b.

The lowest concentration that was introduced to the chamber was ~1 ppm of ethanol, but experimental results showed potential for lower concentration detection because the signal value per ppm was as high as 2.5 mV.

### 3.3. Resolution

**Figure 8:**
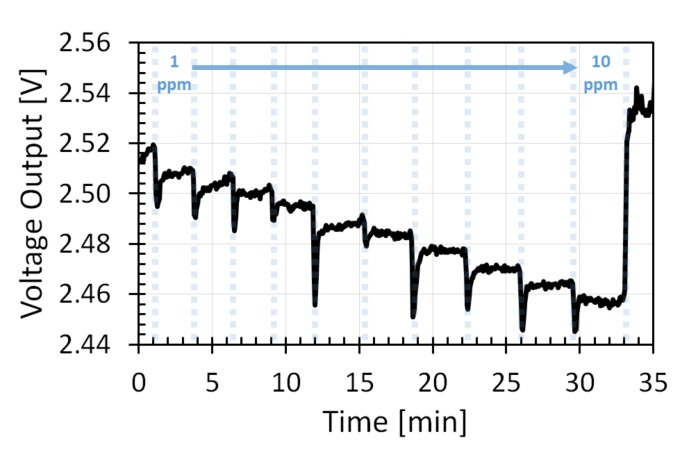
Ethanol measurements at low concentrations over Pt nanoparticle layer. Gradual addition of 1 ppm of ethanol to the chamber from 1 to 10 ppm. Voltage output signal with 20 dB gain at heater-voltage amplitude applied to heating resistor of 3.0 V.

The resolution of the GMOS sensor with a Pt nanoparticle catalyst was estimated by the gradual introduction of gaseous ethanol portions into the gas chamber by a syringe. Each portion added 1 ppm ethanol concentration to the air in the chamber. A signal of 1 ppm was clearly resolved. The voltage signal from each ppm was about 6 mV (Figure 8), meaning that a resolution of less than 1 ppm could easily be obtained. To receive a clear signal for concentrations below 10 ppm, higher catalytic-plate temperature (heater voltage of 3 V) and higher gain (20 dB) are required. A signal at lower temperatures (heater voltage of 1.8 V) could still be observed for concentrations below 10 ppm, but with lower resolution. For the sputtered Pt catalytic layer, a clear signal was not observed at the same conditions, again showing the advantage of the nanoparticle catalyst.

### 3.4. Selectivity

**Figure 9:**
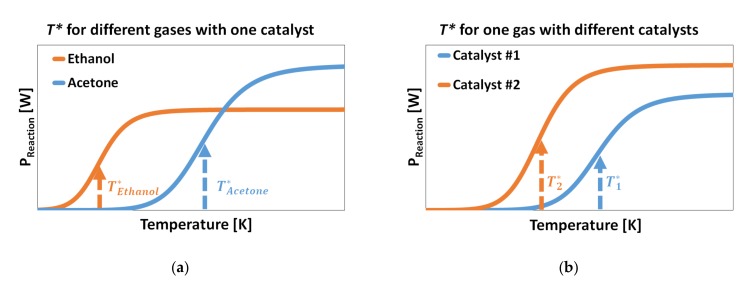
There are two approaches to detect the gases in binary mixtures: using (**a**) a catalytic layer for two different gases or (**b**) a specific catalytic layer for each gas.

Selective sensing is based on the fact that ignition temperature is an inherent property of a catalyst and analyte-gas combination. There are two main approaches [6] for selective gas sensing in mixtures, as described in Figure 9.

The first approach (Figure 9a) uses one catalytic layer for the detection of two different gases. Each gas has a different T* over this catalyst. The initial step is to measure the signal at a lower ignition temperature to detect the first gas and calculate the concentration. In the case of the presence of the first gas, the expected signal response for this gas at a higher ignition temperature can be predicted by having the calibration curve. The second step is to measure the signal at a higher ignition temperature for the presence of the second gas. The output signal at a higher ignition temperature should be compared to the predicted output signal for the first gas. High deviation from the predicted signal is an indication for the presence of the second gas in mixture, and the signal difference corresponds to the concentration of the second analyte gas.

The second approach (Figure 9b) uses a specific catalytic layer for each analyte gas. At each catalytic surface, the analyte gas is measured at the ignition temperature that corresponds to this gas. For this method, two different catalytic layers are required for binary mixture analysis, and the output signal at each catalytic layer corresponds to the specific gas concentration. Both approaches can be applied to the GMOS sensor, having several active pixels and allowing the deposition of different catalysts.

**Figure 10:**
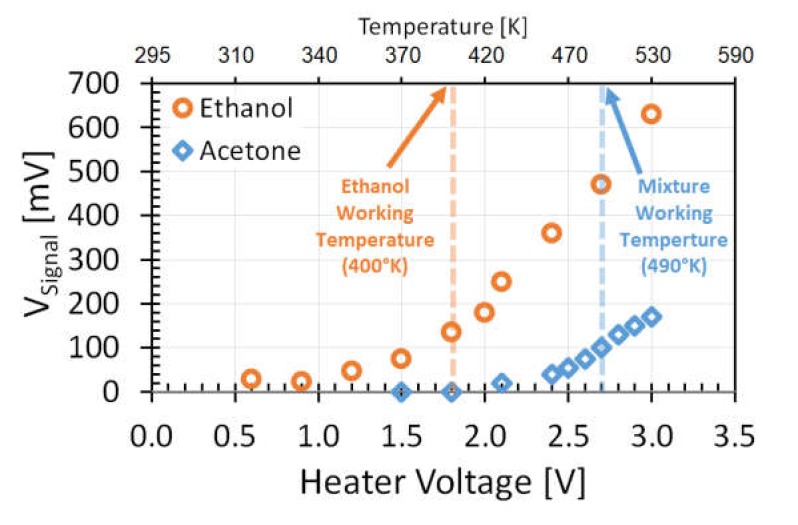
Ethanol and acetone output signal as function of heater voltage applied to heating resistor, and temperature at constant acetone concentration of 100 ppm.

In this study, the first approach is demonstrated by measuring different gases over a Pt nanoparticle catalytic layer at two different temperatures. This approach requires voltage-signal calibration versus stage temperature for each gas and catalytic-layer combination. Such curves for ethanol and acetone are shown in Figure 10; the working temperatures were chosen from these curves. The low working temperature is defined as the temperature where only one gas can be detected. The high working temperature is selected in a way that an adequate signal can be detected from the both gases. Using these guidelines, heater-voltage amplitudes applied to the heating resistor were chosen to be 1.8 and 2.7 V for low and high working temperatures, respectively (dashed lines on Figure 10).

The signal for ethanol on this curve is lower than the signal shown in Figure 7b because sensors in this experiment were used with a mechanical stainless-steel mesh filter on top of the GMOS chip carrier to prevent airflow, which could affect gas measurements. This filter is required for reliable gas detection because gas measurements may also be performed outside the gas chamber. Sensitivity measurements (Section 3.2) were performed without the mesh filter to observe the maximal obtainable sensitivity.

**Figure 11:**
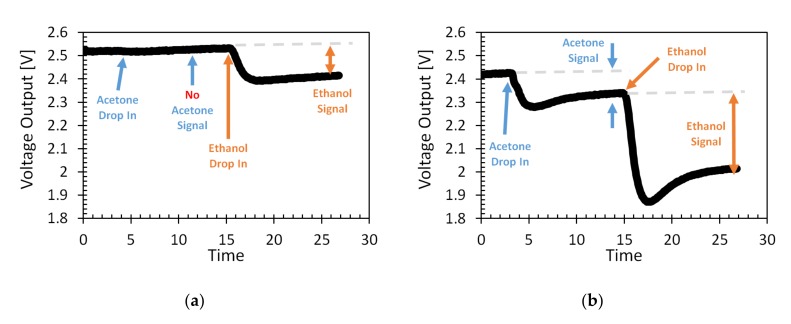
Voltage signal versus time at heater voltage of (**a**) 1.8 V and (**b**) 2.7 V, which correspond to low and high ignition temperatures, respectively. Concentration of each gas was about 100 ppm.

In order to validate gas recognition in the mixture by GMOS, two different gases, ethanol and acetone, were consequently introduced into the chamber, as shown in Figure 11. At a lower ignition temperature (Figure 11a), there was no response for acetone. This temperature was not sufficient for acetone combustion reaction over the Pt nanoparticle catalyst, but it was enough for the detection of ethanol. The same experiment was performed at a high working temperature (Figure 11b). This time, temperature was sufficient for acetone ignition, as can also be seen in Figure 10; therefore, the output signal was clearly observed. After signal stabilization, ethanol gas was introduced to the chamber, and a high signal response was also observed.

These results demonstrated that the concentration of each gas in the mixture could be calculated according to the following algorithm:

(11) $$\left(\begin{array}{l} C_{Gas,1}\\ C_{Gas,2} \end{array}\right)={\left(\begin{array}{cc} S_{11} & S_{12}\\ S_{21} & S_{22} \end{array}\right)}^{-1}\left(\begin{array}{l} v_{sig,1}\\ v_{sig.2} \end{array}\right),$$

where *S*_11_, *S*_12_, *S*_21_, and *S*_22_ are the chemical-sensitivity parameters (V/ppm) that were defined at a low and high working temperature for ethanol and acetone gases, respectively. The methodology presented here may provide a good indication for the presence of different gases in the sensor surroundings. For the exact gas concentration in the mixture, careful calibration work is required.

Future work will focus on the validation of the second approach for selective gas sensing in mixtures by using different catalytic layers for each gas. The combination of the two approaches can provide proper calibration for accurate gas-concentration measurements in mixtures. Environmental issues on sensor performance, such as humidity and temperature effects, will be also studied.

## 4. Conclusions

A novel miniature and selective GMOS gas sensor for gas-mixture analysis was presented. Catalyst-deposition technologies for wafer-level back-end processing were developed. The thermal refreshment of the catalyst with an embedded heater provided long-term sensor operation. As a result, high sensitivity to ethanol for concentrations as low as 1 ppm, with 1 ppm resolution, was achieved. Potential for even lower gas-concentration detection was proved. The feasibility of selective gas sensing in ethanol/acetone mixtures was demonstrated using ignition temperature as the inherent property of the gas/catalyst combination. These advantages make GMOS a good candidate for the next generation of smartphones, wearables, and IoT applications with embedded gas sensors.

## References

[1] Fleischer M., Lehmann M., Fleischer M., Lehmann M. (2012). Solid State Gas Sensors–Industrial Application (Springer Series on Chemical Sensors and Biosensors).

[2] Graf M., Barrettino D., Baltes H.P., Hierlemann A. (2007). CMOS Hotplate Chemical Microsensors.

[3] Hierlemann A. (2005). Integrated Chemical Microsensor Systems in CMOS Technology.

[4] Gardner J.W., Guha P.K., Udrea F., Covington J.A. (2020). CMOS interfacing for integrated gas sensors: A review. IEEE Sens. J..

[5] Jaaniso R., Tan O.K., Jaaniso R., Tan O.K. (2013). Semiconductor Gas Sensors.

[6] Korotcenkov G. (2013). Handbook of Gas Sensor Materials: Properties, Advantagesand Shortcomings for Applications: Conventional Approaches.

[7] Korotcenkov G. (2014). Handbook of Gas Sensor Materials: Properties, Advantages and Shortcomings for Applications.

[8] Ihokure K., Watson J. (1994). The Stannic Oxide Gas Sensor Principles and Applications.

[9] Symons E.A., Sberveglieri G. (1992). Catalytic gas sensors. Gas Sensors Principles, Operation and Developments.

[10] Miller J.B. (2001). Catalytic sensors for monitoring explosive atmospheres. IEEE Sens. J..

[11] Firth J.G., Jones A., Jones T.A. (1973). The principles of the detection of flammable atmospheres by catalytic devices. Combust. Flame.

[12] Riegel J., Härdtl K.H. (1990). Analysis of combustible gases in air with calorimetric gas sensors based on semiconducting BaTiO3 ceramics. Sens. Actuators B Chem..

[13] Pranti A.S., Loof D., Kunz S., Zielasek V., Bäumer M., Lang W. (2019). Ligand-linked nanoparticles-based hydrogen gas sensor with excellent homogeneous temperature field and a comparative stability evaluation of different ligand-linked catalysts. Sensors.

[14] Brauns E., Morsbach E., Kunz S., Bäumer M., Lang W. (2014). A fast and sensitive catalytic gas sensors for hydrogen detection based on stabilized nanoparticles as catalytic layer. Sens. Actuators B Chem..

[15] Gitelman L., Stolyarova S., Bar-Lev S., Gutman Z., Ochana Y., Nemirovsky Y. (2009). CMOS-SOI-MEMS transistor for uncooled IR imaging. IEEE Trans. Electron Devices.

[16] Nemirovsky Y., Svetlitza A., Brouk I., Stolyarova S. (2013). Nanometric CMOS-SOI-NEMS transistor for uncooled THz sensing. IEEE Trans. Electron Devices.

[17] Saraf T., Brouk I., Bar-Lev S., Unikovsky A., Blank T., Radhakrishnan P., Nemirovsky Y. (2016). CMOS-SOI-MEMS uncooled infrared security sensor with integrated readout. IEEE J. Electron Device Soc..

[18] Zviagintsev A., Blank T., Brouk I., Bloom I., Nemirovsky Y. (2017). Modeling the performance of nano machined CMOS transistors for uncooled IR sensing. IEEE Trans. Electron Devices.

[19] Zviagintsev T., Blank I., Brouk S., Bar-Lev S., Stolyarova A., Svetlitza I.B., Nemirovsky Y. Micro-machined CMOS-SOI transistor (TMOS) thermal sensor operating in air. Proceedings of the IEEE COMCAS—International Conference on Microwaves, Communications, Antennas and Electronic Systems.

[20] Nemirovsky Y. (2019). Gas Sensing Device and a Method for Sensing Gas.

[21] Nemirovsky Y., Nemirovsky A., Melman S. (2016). Gas Sensing Device having Distributed Gas Sensing Elements and a Method for Sensing Gas.

[22] Nemirovsky Y., Stolyarova S., Blank T., Svetlitza A., Bar-Lev S., Zviagintsev A., Brouk I. (2018). A new pellistor-like gas sensor based on micromachined CMOS transistor. IEEE Trans. Electron Devices.

[23] Shlenekvitch D., Avraham M., Stolyarova S., Blank T., Nemirovsky Y. Catalytic gas sensor based on micro machined cmos transistor. Proceedings of the 2019 IEEE International Conference on Microwaves, Antennas, Communications and Electronic Systems COMCAS.

[24] Bartholomew C.H., Farrauto R.J. (2011). Catalyst Deactivation: Causes, Mechanisms, and Treatment, Fundamentals of Industrial Catalytic Processes.

[25] Bogue R. (2013). Recent developments in MEMS sensors: A review of applications, markets and technologies. Sens. Rev..

[26] SGX Sensortech. http://www.sgxsensortech.com/.

[27] Magnetron Sputter Deposition System AJA International Inc ATC 2200. http://www.ajaint.com/.

[28] Scienion. https://www.scienion.com/.

[29] Brauns E., Morsbach E., Schnurpfeil G., Bäumer M., Lang W. (2013). A miniaturized catalytic gas sensor for hydrogen detection based on stabilized nanoparticles as catalytic layer. Sens. Actuators B Chem..

[30] Fraunhofer Institute for Ceramic Technologies and Systems IKTS. https://www.ikts.fraunhofer.de/en.html.

[31] Frank-Kamenetskii D.A. (1955). Diffusion and Heat Exchange in Chemical Kinetics.

[32] Schwartz A., Holbrook L.L., Wise H. (1971). Catalytic oxidation studies with platinum and palladium. J. Catal..

